# Collective Prevention of Non-Communicable Diseases in an Ageing Population with Community Care

**DOI:** 10.3390/ijerph20043134

**Published:** 2023-02-10

**Authors:** Regina Kuppen, Mirjam de Leede, Jolanda Lindenberg, David van Bodegom

**Affiliations:** 1 Department of Public Health and Primary Care, Leiden University Medical Centre, Hippocratespad 21, 2333 RC Leiden, The Netherlands; 2 Leyden Academy on Vitality and Ageing, Rijnsburgerweg 10, 2333 AA Leiden, The Netherlands; 3 Buurtzorg Nederland, Head Office, Postbus 69, 7600 AB Almelo, The Netherlands

**Keywords:** collective prevention, public health promotion, community care, social environment, lifestyle

## Abstract

The Dutch population is rapidly ageing, and a growing number of people are suffering from age-related health problems such as obesity, cardiovascular diseases and diabetes. These diseases can be prevented or delayed by adapting healthy behaviours. However, making long-lasting lifestyle changes has proven to be challenging and most individual-based lifestyle interventions have not been effective on the long-term. Prevention programs focused on lifestyle should involve the physical and social context of individuals, because the (social) environment plays a large role in both conscious and unconscious lifestyle choices. Collective prevention programmes are promising strategies to mobilize the potential of the (social) environment. However, little is known about how such collective prevention programs could work in practice. Together with community care organization Buurtzorg, we have started a 5 year evaluation project to study how collective prevention can be practised in communities. In this paper, we discuss the potential of collective prevention and explain the methods and goals of our study.

## 1. Introduction

The world’s population is rapidly ageing, resulting in a growing number of people with a multitude of health problems related to older age [1], such as cardiovascular diseases, diabetes and obesity. Social and psychological aspects such as loneliness and stress are also common among older people and may negatively impact a person’s health and quality of life. Current healthcare systems, which are mainly focused on curative care and acute conditions, are struggling to provide the necessary care for ageing populations [1]. In order to improve the lives of older people and build stronger, healthier societies, The World Health Organization (WHO) has declared 2020–2030 the Decade of Healthy Ageing. Healthy ageing is defined as “the process of developing and maintaining the functional ability that enables well-being in older age” [1]. Functional ability relates to the interactions between people and their environment. Both social and physical environments influence people’s abilities to do the things that are important to them and can improve the quality of life. The action plan for the Decade Of Healthy Ageing requires a society-wide response and emphasizes the need for collaboration with older people, engaging them in the community and create age-friendly, physical, social and economic environments [2].

The above-described paradigm of healthy ageing requires public health policies that focus on preventive care and multidimensional integrated healthcare services instead of the fragmented healthcare designed to cure acute diseases [3]. Beard and colleagues highlight the importance of the shared responsibility of long-term care with families and communities, and of removing barriers to social participation. Such public health policies can find inspiration from countries such as Cuba and Japan where population-wide preventive care has been implemented and the health benefits of a more collective, preventive approach have become evident [4,5]. Cuba, for instance, is a middle-income country with limited resources available for healthcare. At the same time, the life expectancy at birth is almost equal to that of high-income countries. This is considered to be primarily the result of their community-based healthcare approach. The Cuban healthcare system is designed to prevent health problems by focusing on primary care and health promotion in the community [6]. This is illustrated by *Círculos de Abuelos*, a successful long-standing population wide program to promote social and physical activities in older adults [7,8]. The program was founded in 1987 and in 2018 almost 40% of Cuba’s older population participated in the program [9,10]. Other countries such as Venezuela, Panama and Colombia have also started implementing this program. Another example comes from Japan, where the government switched their long-term care prevention from a strategy focused on high-risk individuals to a community-based strategy by organizing ‘salons’: social participation interventions during which older people can gather for enjoyable and educational programs [5]. Participation in these salons was associated with a decrease in cognitive decline and long-term care needs. The Japanese government has also funded Silver Human Resource Centres (SHRC), created by local communities and municipalities, that provide part-time work for people after retirement [11]. These well-established Centres are meant to improve the role and status of older people and help them improve or maintain social participation in the community. The majority of SHRC members are men, for whom working at a SHRC job is associated with increased well-being [12]. This effect was not found for women, possibly due to the fact that more women than men joined for financial reasons. The general motivation for joining a SHRC was psychological growth.

The Netherlands is also taking steps towards a healthier population by focusing more on prevention, as described in the 2018 National Prevention Agreement. This agreement outlines goals for reducing smoking, overweight and excessive alcohol consumption through a healthier lifestyle and changes in the environment such as smoke-free areas, regulations for fewer calories in food, and restrictions on marketing of alcoholic drinks [13]. Building on the goals of this agreement and taking inspiration from the previously mentioned examples from other countries, community healthcare organisation Buurtzorg has taken up the initiative to find new ways of implementing prevention strategies and evaluating its effects on the population in order to contribute to a better healthcare system for the ageing population in the Netherlands. Buurtzorg is a pioneering community care organisation with a nurse-led model of holistic care with self-managing teams of nurses [14]. The nurses help their clients to explore the possibilities to engage their environment and social network in working towards independence and improved quality of life. Buurtzorg’s vision on community healthcare aligns with the WHO healthy ageing framework and with the idea that collective prevention strategies should focus on all the aspects that influence health and well-being, from physical functioning to social environment.

While collective community-based strategies to prevent non-communicable diseases have a large potential, successful implementation in a local context remains difficult in most developed countries [15,16,17]. The diversity in context, design, measurement and evaluation makes it difficult to identify effective community-wide prevention strategies. We need more evidence on and guidelines for effective collective prevention programmes that suit the needs and wishes of the communities and that take the physical and social environment into account. Moreover, we need to further investigate indications for the long-term effects of these programmes on health and well-being of participants. In this article, we describe the study protocol of a Dutch nationwide experiment, developed together with Buurtzorg, with collective prevention strategies in local contexts.

### Background

Lifestyle changes such as adopting a healthy (Mediterranean-type) diet and more physical exercise can prevent or improve many age-related health problems [18]. For cardiovascular diseases, for example, over 70% of the cases and deaths in a worldwide population were attributed to modifiable risk factors, which mainly consisted of metabolic and behavioural risk factors [19]. Type 2 diabetes is another common disease that is related to lifestyle. People with a high adherence to a healthy lifestyle (healthy diet, physical activity, no smoking, weight loss and alcohol-intake) have a 78% less likely to develop type 2 diabetes then people with low or no adherence to healthy lifestyle [20]. Furthermore, up to 40% of dementia cases can be prevented or delayed through a healthy lifestyle [21]. In addition to the earlier mentioned well-known lifestyle factors, social and cognitive factors such as social support, leisure and education also play an important role.

Changing one’s lifestyle is challenging, as there are usually no immediate health rewards. Nonetheless, most preventive lifestyle interventions are targeted at the individual and are not part of regular health care [22,23,24]. While these lifestyle interventions, such as counselling, education, physical training, and cooking workshops may be organized in groups, the responsibility is put on the individual to eat healthily, exercise often, obtain enough rest and maintain other healthy habits in an environment and society that does not promote these healthy behaviours [24,25]. These individual-focused lifestyle programmes have shown to be effective in improving risk factors such as physical activity and bodyweight, but the effectiveness of these interventions is often short-lived and declines with longer follow-up [15,26]. Community engagement has been identified as a key component for maintaining the effects after the end of the intervention [15]. This could be partially explained by the notion that a person’s surroundings and norms and values of people around them have a strong influence on their lifestyle and their ability to change this lifestyle [27,28]. Therefore, it may be more effective to address lifestyle through a collective community-based approach, rather than an individual approach. Within a collective community-based approach, the whole community assumes ownership over a health problem and takes action to address it [29]. Healthy behaviour is best maintained when it fits in people’s daily routine and when it can be combined with something pleasant, such as social relations or activities [27,28]. Individuals with obesity for example can only maintain their weight loss on the long term if they are in an environment that promotes healthy eating and physical activity [24,30,31]. These studies indicate that the social environment is an important factor in health promotion and adherence to a healthy lifestyle.

Many studies indicate the potential of public prevention, but there is still a lack of knowledge about the effective elements of collective prevention in practice and the long-term effects on health and well-being measures [32,33,34]. One important element for collective prevention is investment in community capacity building, as this will lead to more and sustained positive health outcomes [15]. However, as Nickel and Von dem Knesebeck mention [34], in a community there is usually a minority of more active and participative individuals who provide input on the needs and interests of community members, while too little is known about the silent majority. This may lead to over-representation of certain interests, making it difficult to create a program that is supported by all community members. This can affect the effectiveness and sustainability of a collective prevention program.

Collective prevention strategies can take place in collaboration with health care professionals (e.g., general practitioners, physiotherapists, dieticians and district nurses). Especially district nurses (DN) in the Dutch system are familiar with the local context and generally trusted by community members, as they work in close contact with and in the personal environment of their clients. In the Netherlands, district nurses provide care assistance at home. In general, they provide nursing and personal care (e.g., assisting with activities of daily living (ADL), wound care and medication). Their medical expertise and status may contribute to the approachability and willingness of community members to work on a collective prevention project together. Even though there are several collective prevention initiatives in the Netherlands, (e.g., Social Connector [35] as linkage between care, well-being and active citizenship and Community Wise [36] where older members of low SES neighbourhoods took part in 12 active meetings focused on healthy lifestyle) only few can be found to have involved DN [35,36]. An exception is Positive Health in Vensterpolder, where a community care organisation was a stakeholder, but did not have a large role in the project [37]. However, this project turned out to be too much focused on the individual rather than the collective, according to the project leader. There is evidence for nurses having a key guiding or coaching role in lifestyle intervention programmes [38,39].

Thus far, collective prevention initiatives have not often been well documented and studied with validated methods. Furthermore, much research on this subject consists of randomized controlled trials, that are likely too short or not intensive enough to measure change in outcome measures such as participation and quality of life [40,41]. Action research may complement these studies, as community members are involved in the collective prevention project from the start. This may lead to community engagement and projects that are adapted to the local context. In the next section we describe the protocol of our “Collective Prevention” study in which we will evaluate several collective prevention ‘projects’—interventions or activities focused on a healthy lifestyle, social participation, and well-being—designed by DN and community members in their neighbourhoods according to the needs of the community. We will study the effective elements and required motivations and skills from DN for successful collective prevention projects and evaluate the health and social effects of these projects on the community members and the effects on work satisfaction for DN.

## 2. Materials and Methods

### 2.1. Population

The study population in this Collective Prevention project will consist of two groups: DN and community members. The DN are all employees of community healthcare organisation Buurtzorg. Buurtzorg has created a model of care in which self-managing teams of 6–12 nurses deliver all the care that a patient needs. They help people get ready in the morning, wash them, give medication, but also provide medical care such as wound treatment. The teams try to involve the formal and informal networks of their patients to sustain independency for as long as possible. The teams organize their work together and share the workload and responsibility for the quality of care. Buurtzorg has invited all interested teams to apply for the current project by submitting their motivations to work on prevention in their work area. The whole team needs to be on board with the project and they are made aware that they will spend roughly 8 hours a week on the project. The motivations and capabilities of the teams were assessed during an interview. At the time of writing, 18 Buurtzorg teams, from 14 different localities all over the country, are selected to participate. These localities vary in number of inhabitants (1230–19,400), mean income (€26,000–€40,000) and urbanity (1–5) on a scale from 1 (most urban: >2500 addresses per km^2^) to 5 (least urban: <500 addresses per km^2^) [42]. The target population of the collective prevention projects consists of the 55+ population in the neighbourhoods under study. The only strict inclusion criterium for this study is that the participants live in one of the neighbourhoods under study and participate in the project in the neighbourhood on a regular basis. DN will be responsible for recruitment of this study population through interviews and networking in their locality, for which they will receive training and tips during workshops led by the researchers. Participation in both the prevention project and this study is voluntary and, therefore, we work with a convenience sample. Given the range of localities we aim for a diverse population in terms of sex, civil status and income. We expect to involve approximately 25–30 community members per site, amounting to a total of 350–420 individuals in our community sample.

### 2.2. Design

As previous results of population wide prevention have been difficult to establish and this project takes place in the community, we choose an iterative, practice-based design that allows us to make ongoing adjustments during the implementation process. A participatory action research design fits the need for both responsiveness and a contextual approach. We will use mixed methods of data collection to gather insights in implementing public prevention in our healthcare system together with community members and Buurtzorg DN. The aim is that these insights allow all Buurtzorg teams to organise prevention programmes in their localities. Therefore, we will study the motivations of DN to work on prevention and the required qualities and knowledge of DN to successfully establish a prevention programme. The participatory action research approach allows us to immediately respond to the insights we gather during the project, so we can continue to evaluate and adapt the process during the project. For immersive collaboration with DN, every participating Buurtzorg team will choose one team member to be the co-researcher in this project, who will be in regular contact with the action researcher RK. As participatory action research is about doing research *with* instead of *on* people, these co-researchers will have a broad role in the entire project. They will be involved in data collection, reflections on the process of the study and their own experiences, contributing to the projects of other teams by sharing the lessons they learn along the way and helping to interpret the analysed data.

The study will consist of three phases. First, DN of each team will create an overview of their neighbourhood based on their prior knowledge, publicly available data, observations about the physical and social environment, networking with stakeholders and qualitative data gathered through interviews with community members. This overview will provide direction for possible health-related risks or issues in the neighbourhood that might be addressed in a local collective prevention project. In the second phase is the DN will, together with community members, prioritize and decide which subject to focus on. The third phase consists of designing and organising a collective prevention project in the locality together. The collaboration with community members in these steps is key to ensure that the programme is relevant to and created with the community, which are important preconditions for community building.

The time investment for the project as a whole, as well as the possible difficulties or delays in any of the phases will differ between the teams. Therefore, we use a stepped wedge design in this project. We will follow the teams and their prevention projects for 4–5 years, at the end of which we will be able to understand how collective prevention projects can be developed in collaboration with DN and community members, what the effective elements and obstacles are, and how it affects public health. The multimethod design ensures a rich and diverse representation of the impact of the collective prevention projects on the target audience. The quantitative effect measures will provide an indication of the change over time and possibly between groups. The qualitative measures in this study will provide a more detailed and personal image of health, well-being, motivations and other experiences during the project, as well as insight into elements that matter in the process. This will help to determine which projects can be considered successful and how they might be implemented on a larger scale.

### 2.3. Procedure

In the learning process of participative action research, qualitative methods can provide context specific and detailed information from the different perspectives of participants. Therefore, we keep the number of questionnaires to a minimum. Furthermore, long questionnaires can discourage community members from participating in the study, due to the time investment and possibly a perceived breach of their privacy. The general attitude of DN towards questionnaires is not positive either. They indicated that they prefer personal and informal communication with community members to build and maintain a relation of trust. After discussion, we have selected three questionnaires amounting to 18 questions in total and a selection of 17 questions from an annual employee questionnaire from Buurtzorg. We will start with a baseline measurement at the start of the project and repeat the data collection annually for four years, resulting in a total of four measurement times during the project.

During these four years, the researcher will conduct a number of interviews and focus group discussions to evaluate the process and gain insights in the experiences the DN and the community members during the project. These meetings will be voice- and/or video-recorded after permission from the participants. Observations about the atmosphere, non-vocal communication and other contextual information will be noted. In addition to the standard evaluation and measurements for every prevention project, we may deploy other methods for project specific outcomes in collaboration with the co-researchers. This will ensure that the methods suit the specific projects and their envisioned targets and that co-researchers are satisfied with the way their projects are evaluated.

Throughout the whole project, Buurtzorg teams will receive support from one of the three Buurtzorg coaches who are part of the project team for the organisation of the Collective Prevention programme. Additionally, the researcher will have monthly contact with the co-researchers to gather experiences from the team and evaluate the process of developing a collective prevention project. Finally, (participatory) observations will be made throughout the duration of the study, for example during brainstorm sessions, meetings or activities of the prevention projects.

In the first phase, the DN will gather information about people’s social situation and common health issues in the neighbourhood where they work by conducting at least 30 interviews with community members and health professionals such as general practitioners, physiotherapists and dietitians. DN will receive instructions and training for conducting interviews during webinars and workshops. The goal of these interviews is to determine what community members of every participating neighbourhood consider important aspects of public health in their lives and environment that may be improved by a prevention project. During this phase, the Buurtzorg teams will also ask permission and gather contact information from community members (and other stakeholders) who are willing to participate in this study. The researcher or co-researcher will explain the purpose, procedure, potential benefits and risks of participating in this study orally and in writing. We will also gather statistics about health problems and other relevant factors in the neighbourhood via publicly available data (such as the National Institute for Public Health and the Environment (RIVM) or Statistics Netherlands (CBS)). Based on this analysis, Buurtzorg teams together with community members will identify the topic for their prevention programme and develop a plan of action by again conducting (group) interviews and using design methods that will be discussed during monthly webinars.

### 2.4. Impact Measurement

**Figure 1:**
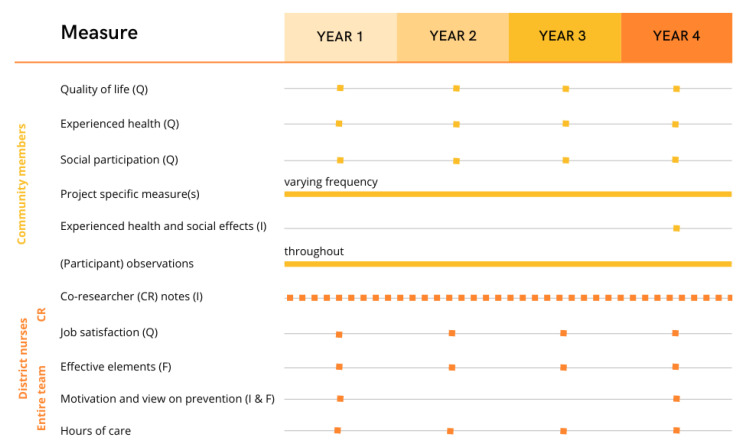
Impact measurements of collective prevention projects. Note: Q = questionnaire, I = Interview, F = focus group. CR = Co-researchers: the district nurses who have an active role in the development of the prevention projects.

An overview of the study design and the different measures in the study protocol can be found in Figure 1.

#### 2.4.1. Community Members

##### Health and Well-Being

Health and well-being of the community members will be evaluated by measuring two variables—quality of life and experienced health—annually in each project. Quality of Life will be measured using the validated Dutch ICECAP-O, the ICEpop CAPability measure for Older people, that consists of 5 items on a 4-point scale [43,44]. We use this measure that is specifically developed for older adults, because we expect that the majority of our study population will be older adults. Moreover, we expect that a capability approach, mapping both the capabilities in the environment as well as the degree to which members can take up these capabilities fit with the environmental approach of collective prevention. Experienced Health will be measured with the 4-item index of Perceived Physical Health [45]. These items are scored on a 5-point scale. The questions of this measure were translated from English to Dutch for the current project. We will use the WHO methodology for translation and adaptation of instruments to translate this instrument. In this methodology, we will translate and backtranslate the instrument after which we will pilot test it before using it for data collection in the whole study [46].

In the last year of the study, we will interview community members who participate in the prevention projects to gather qualitative data about health and social effects, their experience with the prevention projects and how it may impact their daily life. We will pose questions such as “What does preventive care mean for you?”, “How has the prevention project impacted your physical functioning, condition or energy level?”, and “What changes have you experienced in your social life or network since participating in this project?” (see Supplementary Materials, Topic list S1). Additional data about community members’ experiences in this project may come from (participatory) observation sessions throughout the 4 years of this study.

##### Social Participation

To study the social effects of the prevention projects and because of the focus on community engagement and building a social network in this project, we measure social participation. We chose this over other measures of loneliness or social isolation, because it is a broader outcome variable of social connectedness that participants may be more willing to talk about. We will measure social participation with a questionnaire consisting of 9 items from the participation section of the Social Domain Index. This is a Dutch survey from The Netherlands Institute for Social Research (SCP) that is used to measure national trends [47]. It consists of questions about participation in any clubs or associations, sports, volunteer work, theatre and more (see Supplementary Materials, Questionnaire S1). Four of these items are scored on a 4-point scale, 3 items require a yes/no answer and 2 items an estimate of the hours spent on a certain activity.

##### Additional Project Specific Variables

In addition to these annual, general measures, we will use measures dependent on the specific topic of the local prevention project. Depending on the outcomes in phase 1, the projects could focus on lifestyle or social connectedness and measures added could include variables such as loneliness, mental well-being and physical condition. The methods to evaluate these variables will be determined together with the co-researcher of the team and may consist of validated questionnaires measuring a certain variable (e.g., Lubben Social Network Scale), interviews, focus groups or other qualitative methods of obtaining data, for example through photo voice, elicitation or narrative techniques.

#### 2.4.2. District Nurses

##### Job Satisfaction and Motivation

The impact on job satisfaction of the DN will be evaluated using a selection of 17 out of 77 questions from the standard annual Buurtzorg job satisfaction questionnaire (see Supplementary Materials, Questionnaire S2). This selection contains the questions that are most relevant to job satisfaction in relation to this project. The DN of the participating Buurtzorg teams who have a key role in the development of the prevention project will complete this questionnaire once every year. We will also obtain mean scores on these 17 items of Buurtzorg employees who do not participate in the study. This will provide us with a control measure, so we can compare the change over the course of this study between the involved nurses in our programme and those involved in regular care of Buurtzorg. Similarly, we will collect data that Buurtzorg has on mean hours of care per client to track the changes during our project and compare the participating teams with regular teams.

The researchers will conduct individual semi-structured interviews in the first and last year of the project to gather information about DN’s views on preventive care, their motivations to work on prevention, the relation to community care and their view on the prevention project in their team. We will ask questions such as “How does prevention fit into the work of DN according to you?”, “How was the subject of prevention represented in your education and how does this relate to your current view on prevention?”, “Can you name some positive and some less positive aspects of your team’s collective prevention project?” (see Supplementary Materials, Topic list S2). These interviews will be followed by focus groups with each Buurtzorg team with a topic list that transpires from the outcomes of the individual interviews. Topics that are likely to be discussed are different views on prevention, representation of prevention in education, roles in and views on the Collective Prevention programme and collaboration within the team. Prior to these interviews, we obtained informed consent from the DN.

### 2.5. Process Evaluation

#### Effective Elements

During the entire project, we will gather data on the process of developing a collective prevention project to determine effective elements. This will be achieved in regular meetings between researcher and co-researchers. These informal conversations are designed to keep track of each team’s progress and to ask the co-researchers about breakthroughs, questions or possible issues they encounter in their project. This provides input for topics or information that are addressed in webinars with the larger group of participating DN. These webinars will take place once per two months in the first year, and approximately once per three months in the following years. Furthermore, the researcher will visit relevant events or activities of the prevention projects, such as walking groups, workshops or other gatherings, to gather observational data in the form of notes. Lastly, annual focus groups will be held with DN based on a semi-structured topic list (see Supplementary Materials, Topic list S3). This list consists of the following topics: views on prevention, collaboration with and involvement of community members, process of organising a prevention project and the support that teams receive in this process.

### 2.6. Analyses

#### 2.6.1. Quantitative Analyses

For the quantitative analysis we measure three dependent variables (quality of life, experienced health and social participation) in all of the 14 prevention programmes once a year. We estimate that 25–30 community members will be involved per locality, resulting in a sample size of 350–420 individuals. All variables will be analysed descriptively per year. We will use a repeated measures MANOVA to assess the change within subjects for the dependent variables over time (4 time points). We will use the same test to make between-group comparisons of the individual prevention programmes if the sample size allows this.

Quantitative project specific measures will most likely have an inadequate sample size for a solid quantitative analysis, so these will only be analysed descriptively. In case of multiple teams using the same project specific measure (e.g., Lubben Social Network Scale), we may be able to make comparisons in change of these variables over time and between the different projects. As Buurtzorg teams will determine the design of their project depending on the findings in phase 1, we cannot determine this in advance.

Job satisfaction will be analysed descriptively every year. The change over the years (4 measurement times) will be compared within subjects using a repeated measures ANOVA. We will also conduct a t-test to compare the change in job satisfaction for participating DN with the larger group of Buurtzorg DN who do not participate in the study but answer the same questions as part of the standard annual Buurtzorg questionnaire. The same analysis will be performed on the mean hours of care per client per Buurtzorg team, except in this case each data point represents a team instead of an individual.

#### 2.6.2. Qualitative Analyses

The qualitative data about the impact of the prevention programmes on health and well-being of community members, gathered during the focus groups, interviews and observations, will be transcribed and analysed using the qualitative data software programme MAXQDA (https://www.maxqda.com/, accessed on 8 February 2023). In order to account for subjectivity and personal errors, two researchers will be coding and analysing the data. We will determine which aspects of community members’ health, well-being, social and daily life are linked to participation in the prevention project by conducting an initial explorative content analysis, determining themes, followed by a more in-depth thematic content analysis. Observations and memos made during interviews about any contextual information (e.g., atmosphere) will be discussed between the interviewers and coders.

The analyses for the process evaluation, with the aim of determining effective elements, will be focusing on the observational data and data from focus groups and interviews with DN and community members. This analysis, as well as the analyses of DN’s motivation for and perspective on preventive work will be performed in the same way as described above.

### 2.7. Ethics

This study was reviewed and declared not subject to the law on research involving human subjects by the Institutional Review Board of the Medical Ethical Committee Leiden-Den Haag-Delft for observational studies and was registered under number WSC-2022-10. The protocol is currently under review for compliance with scientific due diligence. Prior to participation in this study, participants will be informed about the goal, procedures, confidentiality of data and other relevant information both orally and in writing. They are then asked to sign an informed consent form for the specific parts of the study that they will participate in. Results and conclusions from the collected data will be given back to Buurtzorg anonymously and will be used to adjust aspects of this study and future prevention projects where necessary. Data will be stored securely on a server at the Leyden Academy on Vitality and Ageing. Randomly generated codes will be assigned to the data, making sure that no personal data can be traced back to responses on questionnaires, interviews or other collected data. These codes are stored in a key separate from the data files and safeguarded through data protection measures. The key can only be accessed by the researcher and two coordinators of this project. The handling of data will comply with the EU General Data Protection Regulation and the Dutch Act on Implementation of the General Data Protection Regulation.

## 3. Discussion

The primary objectives of the described study in this article are (1) to study how collective prevention programmes in community-based healthcare, mainly focused on older people, affects participants’ health and well-being and (2) to evaluate and clarify what it takes for DN to develop and implement such programmes in collaboration with community members. This identifies required skills, knowledge and other essential elements during the programme’s development and implementation. A secondary objective is to gather information about how DN view prevention as a part of their job and what motivates them to work on preventive care. The results of this study will contribute to the discussion of how preventive care fits into our healthcare system in order to make it more suitable for an ageing population. Since a large part of the population encounters the same lifestyle-related health problems at later age, partly due to influences of the environment they live in, this collective approach adapted to the local context is reasoned to be more effective on the long term than healthcare strategies that mainly focus on the individual. Moreover, the fact that this study involves 14 different locations, with variations in number of inhabitants, socio-economic status and urbanity, provides opportunities to compare health effects and effective elements of collective prevention in different contexts.

### 3.1. Implications

This study will provide information about which aspects of health, well-being and social life are affected by different collective prevention projects. As community members will be working together to organise activities, we expect that the impact of the prevention projects for community members will initially be measurable with our social effect measures. Initial participation may be a difficult step for many people, but once people are part of a group, it will be easier to take the initiative of going to other activities or outings in their daily life. This is expected to prevent or decrease loneliness and increase social participation, which has been shown to increase health and well-being [5,48,49]. The relations between people should be carefully considered and monitored to avoid negative effects on well-being due to failure of support, neglect or misdirection, which could lead to outcomes such as increased distress or decreased self-worth and self-efficacy [50]. The goal is to form a local social network with people helping and motivating each other and contributing to collective health goals by making use of each other’s talents. Ultimately, this may also lead to a decreased need for long term care or certain home care tasks. A stronger social network will also mean that people likely have better access to and knowledge of the available support systems and services, which will eventually lead to decrease in the workload of DN. This may contribute to better health care for the entire ageing population, because more people are able to maintain good health for a longer time with no or minimum help from DN. Consequently, DN can help more people without increase in the work hours.

All the prevention projects will be focused on a health-related issue or concern that Buurtzorg teams have identified together with community members. Therefore, this project is expected to improve health in the community. After identifying successful elements in the programme and viable projects, it could be implemented in other locations. It is a particular ambition of the study to use the lessons learned to implement collective prevention in all Buurtzorg teams (N = 950) in the Netherlands. Finally, the project aims to provide evidence for effective and sustainable bottom-up health care for other home care organisations and contributing to future-proof health care systems that fit an ageing population.

The development and implementation process can be time consuming for DN and will include tasks that normally lie outside the scope of their daily work. Therefore, we expect to encounter unforeseen difficulties and challenges along the way. It may also lead to fluctuations in work satisfaction during the project. The population of DN in this study is highly motivated to work on prevention, so we expect that their work satisfaction will eventually increase once the prevention projects are well underway. The effect of motivation patterns on success will also be studied, which can be used as a guideline or criterion for starting a prevention project in a certain team of DN.

We also aim to shed light on other working elements such as required skills, knowledge and appropriate steps to take when organising a collective prevention project. As we expect community engagement to be an important factor in each projects’ success, we will compare our results with the conceptual model for community engagement and pay special attention to elements such as organizational partnerships and alliances [51]. While these partnerships are not specifically assessed in this project, we acknowledge their importance and would suggest more detailed investigation on this topic for future steps of this project. We aim to create a general timeline and guidelines to make implementation of collective prevention programmes possible on a broader scale.

### 3.2. Limitations

This study will take place in 14 different localities with prevention projects that are designed for that specific context. This makes the projects relevant for the neighbourhood, but also comes with challenges in generalisability and comparing the prevention projects and their health and social effects. For example, the effects could be different for different ethnic groups due to underlying differences in views and perceptions regarding health, social norms and collective prevention. This should be taken into account if prevention projects are deployed in more localities. Additionally, the participating Buurtzorg teams are not selected at random, but they apply for participation in the project and are selected based on a strong motivation and willingness to work on prevention. This means that the sample of district nurses in this project likely already has a certain set of characteristics or values that are different from a randomized sample. It should, therefore, be noted that the implementation of collective prevention programmes in all Buurtzorg teams may shine light on new challenges or required skills and knowledge that may have been taken for granted in the selected sample of the current study. Furthermore, it is difficult to accurately measure prevention, as the desired outcome is a non-event. In addition, there is no control group in this study and we can only compare with general population outcomes. Furthermore, we are not able to control for the effect of planning and organizing a project in a community. Structural Governance and Collective Empowerment are two practices in community-based participatory research that influence the outcomes through interaction with the context, relationships and other aspects of the research [52]. This means that the health and social effects that we will measure cannot only be attributed to the content of the prevention project. Finally, results in this study will mainly be based on a population of relatively older people, since they are more likely to have the time to help organise such a project. Whereas collective prevention should be focused on all ages, as this is the best way to collectively change our lifestyle and prevent age-related diseases. This may be a next step for spreading the prevention projects of this study.

## 4. Conclusions

Societal trends, population ageing and the rising rates of chronic conditions warrant the study of new models for health promotion and prevention. The collective model that we will study in this participatory action research will provide a wide range of information about the implementation and effects on the target audiences of collective prevention with regard to the local physical and social environment. This will help to improve the practice and health effects of collective prevention in order to shape a healthcare system that supports and stimulates healthy ageing. In this study, we will evaluate programmes in The Netherlands, but the effective elements from these programmes and the design of this research project may provide a first step for implementation of this healthcare strategy in other countries as well.

## Supplementary Materials

The following supporting information can be downloaded at: https://www.mdpi.com/article/10.3390/ijerph20043134/s1, Topic list S1: Interviews with community members; Questionnaire S1: Social participation questionnaire (Social Domain Index); Questionnaire S2: Job satisfaction questionnaire; Topic list S2: Interview with district nurses: motivation and view on prevention; Topic list S3: Focus group discussions process evaluation.
