# Supplementary material for: Collective Prevention of Non-Communicable Diseases in an Ageing Population with Community Care
Source: Int J Environ Res Public Health. 2023 Feb 10;20(4):3134. doi: 10.3390/ijerph20043134 (PMC9961588; doi:10.3390/ijerph20043134)
Supplement: Supplementary file 1 [file ijerph-20-03134-s001.zip › ijerph-1968710-supplementary.pdf]

Topic list S1: Interview with Community Members

Note: This is a CONCEPT topic list. Questions may be changed, removed or added during the course of the research project to ensure that it is suitable to the projects and participants at that time.

ENGLISH

Introduction

- Can you introduce yourself?
- How long have you lived in this neighbourhood?
- How do you like living here?
- Can you tell me something about your daily life?
- What gives you energy?

Motivation for participation

- How did you get introduced to the prevention project in your neighbourhood?
- Can you tell me what the reasons were for you to participate in this project?
- What did you expect the project to bring you?
- How often in a month do you go to gatherings or activities of this project?
- In what ways are you involved in this project? [only visits, co-creating, organising,…]

Vision on prevention

- What does prevention mean to you?
- Can you tell me something about how age does or does not play a role in the approach of prevention?
- What do you hope to achieve with prevention activities?
- How does prevention already play a role in your life?

Experiences with the prevention programme

- Can you tell me something about your experiences with participating in the prevention programme?
- What are positive aspects of the programme?
- What are things you can appreciates less about the programme?
- Can you tell me something about how the prevention programme has changed things in your (daily) life and functioning?
- What changes do you notice in how satisfied you are with your life since participating in the programme?
- Can you say something about physical changes, such as physical functioning, fitness or energy?
- How do you think that participating in the programme has influenced your mental wellbeing?
- How does the programme contribute to things that are important to you and give meaning in your life?
- What changes have you experiences in your social life since participating in the programme?
- How does the programme contribute to public health in the neighbourhood according to you?
- What do you think about the role that the district nurses have in this programme?
- What would you think about other (health)professionals getting involved in the programme?
- What advice would you give to further improve the programme?

Questionnaire S1: Social Participation Questionnaire (Social Domain Index)

ENGLISH

Introduction

The following topic is leisure activity. Can you indicate how often you have participated in the following activities in the past 12 months?

Association

1. Participating in activities related to an association or club like sports, singing, dance, music, theatre or crafts.

1. At least once a week
2. At least once a month, but not weekly
3. Less than once a month
4. Seldom or never

Theatre

2. Visits to a theatre, concert, cinema, museum, restaurant, cafe or sports match.

1. At least once a week
2. At least once a month, but not weekly
3. Less than once a month
4. Seldom or never

Community centre

3. Visits to a community centre, social centre or service centre.

1. At least once a week
2. At least once a month, but not weekly
3. Less than once a month
4. Seldom or never

Volunteer work

4. Do you work as a volunteer, meaning unpaid work for or organised by an institution or association?

- Yes
- No

5. Can you give an estimate of how much time you spend on volunteer work? You can give an estimate of average amount of hours per week or in total over the year. Please give your answer in hours: 45 minutes should be rounded up to 1 hour.

- [1…997] hours per week
- [1…997] hours per year

Help to the sick or disabled

6. Do you provide uncompensated help to sick or disabled family members, friends, acquaintances or neighbours?

- Yes
- No

7. Can you give an estimate of how much time you spend providing uncompensated help to sick or disabled family members, friends, acquaintances or neighbours? You can give an estimate of average amount of hours per week or in total over the year. Please give your answer in hours: 45 minutes should be rounded up to 1 hour.

- [1…997] hours per week
- [1…997] hours per year

Sports

8. Did you participate in any sports in the past 12 months?

- Yes
- No

9. How often do you participate in sports on average?

1. At least once a week
2. At least once a month, but not weekly
3. Less than once a month
4. Seldom or never
5. I don't know

Questionnaire S2: Job Satisfaction Questionnaire

ENGLISH

1. I am satisfied with the work I get to do for Buurtzorg.
2. I commit to continuously improving myself.
3. It is clear to me which tasks I have to do in my function.
4. I know which results are expected of me.
5. I can deal with changes very well.
6. I enjoy my work.
7. I am proud of the work I do.
8. My work gives me energy.
9. I experience a good work-life balance.
10. I am satisfied with my colleagues.
11. I feel safe enough to openly discuss a mistake I made.
12. I am satisfied with the division of tasks between colleagues.
13. I feel appreciated by Buurtzorg.
14. My organisation is open to suggestions and new ideas.
15. I am satisfied with my development opportunities with Buurtzorg.
16. I want to improve and develop myself in my discipline.
17. In the past 3 months I have taken specific actions to find other work, or am going to do that in the next 3 months.

Response scale for all items:

1 = completely disagree, 2 = disagree, 3 = neutral, 4 = agree, 5 = completely agree

Don’t know/no experience

Topic list S2: Interview with District Nurses: Motivation and View on Prevention

Note: This is a CONCEPT topic list. Questions may be changed, removed or added during the course of the research project to ensure that it is suitable to the projects and participants at that time.

ENGLISH

Introduction

- Can you introduce yourself?
- How did you come to work for Buurtzorg?
- Can you tell me something about your previous work experience?

Task description district nurse

- How did you get interested in becoming a district nurse?
- What does an average day as a Buurtzorg district nurse look like?
- What are the tasks/responsibilities of a district nurse according to you?
- How does this compare to the work you currently do?
- What aspects of your work as a district nurse give you the most energy?
- What gives you the least energy or even takes energy?

Vision on prevention

- What does prevention mean to you?
- How do you think that prevention fits into the responsibilities and tasks of a district nurse as you previously described?
- How is prevention regarded and does it play a role in your team?
- What is your attitude towards preventive work?
- What makes working on prevention worth it according to you?
- What would you need to be able to work on prevention?
- What do you think are possible challenges you would encounter?

Education

- How was prevention addressed in your training/education to become a district nurse?
- How does this compare to how you think about prevention now?
- How do you look back on the way prevention was addressed in your studies?
- What would be your advice to the nursing education programme?

Prevention programme in your neighbourhood

- In what ways are you involved in the prevention programme in your neighbourhood?
- What are your thoughts about the prevention programme in your neighbourhood?
- Can you tell me about some changes you see/have seen in the neighbourhood since the start of the prevention programme?
- What do you think are the most important reasons for community members to participate in the prevention programme?
- What are some positive aspects of the prevention programme according to you?
- What are lesser aspects of the programme according to you?
- What do you think about the role that the district nurses have in this programme?
- Why would you or wouldn’t you want to have this role yourself?
- What would you do differently?
- How do you see a possible role for other (health) professionals in this programme?

- How has your view on prevention changed since the start of the prevention programme?

Collaboration

- What would you like to change about the way that you work on prevention in your team?
- What kind of opportunities do you see for collaboration with other (healthcare)organisations on the subject of prevention?

Topic list S3: Focus Group Discussions Process Evaluation

Note: This is a CONCEPT topic list. Questions may be changed, removed or added during the course of the research project to ensure that it is suitable to the projects and participants at that time.

ENGLISH

Prevention level

- Can you describe how preventive care fits into the work of district nurses?
- What do you think is necessary to achieve the implementation of prevention in your job?
- Why is it important to you to work on collective prevention in the neighbourhood?
- What motivates you to work on this?

Participants level

- What kind of group of participants are you reaching with your project?
- Why do you think you are specifically reaching these people?
- What are motivations for community members to participate in the project as far as you can tell?
- In what ways are community members involved in the activities of the project?
- Can you tell me something about how community members are encouraged to take tasks upon themselves for the organisation of the project?
- How is the general atmosphere or mood in the neighbourhood now compared to a year ago?
- How has the interaction between community members changed since the start of the project?
- What kind of change do you notice in the social lives of the community members?
- How do you make sure that the project suits the needs and wishes of the community members?
- How do you experience the collaboration with community members in this project?
- What kind of role do practical aspects such as location, time or frequency of activities play in the project?
- Which group of people are you currently not reaching with this project that you would still like to involve?
- Can you speculate about the reasons you are not reaching these people right now?

Programme level

Together we established a few outcome measures and aims, of which wellbeing and prevention of disease are the most important ones. The three outcomes that are measured in every prevention programme are experienced health, quality of life and social participation. We would like to hear your thought about these outcome measures, your working methods and how these things may have changed since the start of the project.

- What are the most important outcome measures or results for your programme according to you?
- What would be a way to see or measure that a programme is successful according to you?
- In what ways are you working on prevention in your programme?
- What do you think are the most important aspects to ensure that you keep the programme on the rails?

- What do you like about working on the prevention programme?
- What is challenging about working on the prevention programme?
- Where or to whom can you go for advice or support during this project?
- What are your thoughts about the role/support from Buurtzorg in this?
- What are some things that can be improved in this programme according to you?
- How do you see the future of the programme?
- What role do you see for yourselves as district nurses in this programme?
- What role do you see for other (healthcare) professionals in this programme?
- What opportunities do you see for development of the programme in the neighbourhood?
- What opportunities do you see for development of the programme outside the neighbourhood?

Project level

Lastly, we want to discuss the Collective Prevention project in general to evaluate the support, information and structure of the project so far.

- What do you like about participation in the Collective Prevention project?
    - Knowledge, skills, financing, hours?
    - Expert coaching, support?
    - Exchange between the different teams and programmes: peer coaching and information exchange?
- What are aspects that can be improved in the Collective Prevention project?
- What would be your advice to improve the project further?
